# Does Consumer Confidence Forecast Household Saving and Borrowing Behavior? Evidence for Poland

**DOI:** 10.1007/s11205-016-1376-4

**Published:** 2016-06-10

**Authors:** Aneta Maria Kłopocka

**Affiliations:** University of Finance and Management in Warsaw, Pawia Str. 55, 01030 Warsaw, Poland

**Keywords:** Consumer sentiment, Household saving rate, Household debt, Financial expectations, Subjective and objective indicators, E27, E21

## Abstract

Consumer confidence plays an important role in households’ decision-making processes. This study investigates the effects of consumer confidence on household saving and borrowing behavior that are unsatisfactorily considered in previous discussions. The questions of interest are first, whether indexes of consumer confidence have any predictive power on their own for future household saving and borrowing rates, and second, whether they contain information about future household saving and borrowing rates aside from the information contained in other available indicators. In addition to aggregate confidence indicators, their components are used to provide more precise information. Overall, the multiple linear regression analysis (OLS technique) of Polish time-series data gives positive answers to both questions. This finding supports the recommendation of combining the strengths of objective indicators (such as economic fundamentals) and subjective indicators (such as consumer confidence) to improve household financial behavior forecasts.

## Introduction

The financial stability of an economy is significantly influenced by the evolution of household financial behavior. The financial turmoil that started in 2007 and the severity of the recession that followed highlights the importance of household financial stability as a key factor affecting economic growth. The appropriate shaping of household balance sheets is important on a macroeconomic scale and from the perspective of individual entities. It protects households against possible insolvency and its adverse socioeconomic consequences. Household saving and borrowing decisions demonstrate preferences concerning intertemporal choice. Allocating consumption in time, households reduce (or increase in the case of negative net savings) their exposure to liquidity risk and modify their ability to withstand financial shocks.

Consumer sentiment^1^ plays an important role in households’ decision-making processes. The aim here is to gauge the extent to which confidence indicators (namely, the Current Consumer Confidence Index, the Forward Consumer Confidence Index and their underlying components) have predictive power in explaining aggregate household propensity to save and borrow using time-series data for Poland. Furthermore, the regression analysis focuses on whether confidence indicators contain any information beyond economic fundamentals. The explanatory variables that we treat as “economic fundamentals” are variables usually found to have some predictive power to explain changes in consumption. They include real household disposable income and Monetary Financial Institutions interest rates.

This study relates to at least three strands of the literature. The first identifies the relationship of household financial behavior and economic cycles. Nofsinger (2012) describes household behavior in boom and bust economic cycles, focusing in particular on the recent financial crisis. He reveals that behaviors are motivated by cognitive limitations and psychological bias. Extrapolation bias, groupthink, and changing social norms play an important role. He demonstrates that household behavior exacerbates the boom/bust economic cycle. In boom times, the increase in debt load and decrease in saving rate spur economic growth. In bust times, households repay debt and save more, which drags on an already slow economy. In addition, households influence businesses and governments into actions that also foster the cycle. Kośny (2013) analyzes micro data on changes in the level of savings of Polish households in successive sub-periods and provides evidence that savings increase in periods of slower economic growth and decrease in fast growth periods. He suggests that a possible explanation of this phenomenon may be the importance of precautionary saving.

The second vast strand of literature stresses the influence of uncertainty on consumption and saving. The precautionary motive (“to build up a reserve against unforeseen contingencies”) has assumed the central place in the literature on household saving. Browning and Lusardi (1996) review the empirical evidence on precautionary saving and summarize it as follows: “it seems to us that precautionary motive has some role to play in explaining saving behavior but it is unlikely to be as important as some studies suggest”. Overall, the “precautionary saving” hypothesis has been extensively tested in the literature, and there is abundant evidence that increased uncertainty causes greater savings rates. The most recent examples are those of Carroll et al. (2012), Mody et al. (2012), Bande and Riveiro (2013), Ceritoglu (2013), Chamon et al. (2013), and Mastrogiacomo and Alessie (2014). However, some research finds little or no evidence on the precautionary motive (e.g., Fossen and Rostam-Afschar 2013).

The third strand of literature discusses the importance of consumer confidence in stimulating economic activity. Most studies have focused on the time-series relationship between aggregate consumption and the aggregate indices of sentiment and, in particular, on the question of whether consumer confidence forecasts consumption. The results on the predictability of consumer attitudes toward consumer spending are somewhat mixed. The effect of consumer sentiment on consumption has been analyzed by, among others, Carroll et al. (1994), Kwan and Cotsomitis (2004), Ludvigson (2004), Easaw et al. (2005), Kwan and Cotsomitis (2006), Malgarini and Margani (2007), Celik and Ozerkek (2009), Özerkek and Çelik (2010), Bruno (2014), Lachowska (2013), and Lahiri et al. (2015). Most of these studies, but not all, have focused on the USA. Their results can be construed as supporting the hypothesis that consumer confidence contains information relevant to predicting spending, independent from other indicators, and improves the accuracy of consumption forecasts. Howrey (2001) and Dees and Brinca (2013) show that the contribution of confidence in explaining consumption expenditures increases when household survey indicators feature large changes; thus, confidence indicators can have some increasing predictive power during periods associated with high consumer confidence volatility. Taylor and McNabb (2007) demonstrate that consumer (and business) confidence indicators are procyclical and generally play a significant role in predicting downturns. Christiansen et al. (2014) conclude that sentiment variables hold vast predictive power for US recessions in excess of both the classical recession predictors and the common factors. Conversely, Fuhrer (1993), Fan and Wong (1998), Goh (2003), Cotsomitis and Kwan (2006), and Al-Eyd et al. (2009) suggest that confidence effects on consumption are weak when other key determinants of consumption are considered.

Surprisingly little attention has been directed to the individual component questions that the aggregate consumer confidence indexes are based on. Bram and Ludvigson (1998) undertake a formal statistical comparison of the predictive power exhibited by the University of Michigan’s Consumer Sentiment Index and the Conference Board’s Consumer Confidence Index and their component questions for several categories of consumer spending growth. Their results show that some survey questions have more predictive power than others. Questions that ask about consumers’ perceptions of job availability typically have the most explanatory power for future movements in consumption, whereas questions that ask about buying conditions or financial conditions today relative to the past appear to have much less explanatory power. Wilcox (2007) demonstrates that the individual component questions that comprise the University of Michigan’s Consumer Sentiment Index often much more significantly improve consumption forecasts than does the aggregated index that is constructed from those questions. He reveals that forecasts, not just of durables—or vehicles in particular, but also of nondurables and services are improved by including individual component questions about consumer sentiment. Kellstedt et al. (2015) find that, at least with respect to consumer spending on durable goods, the multi-indicator Index of Consumer Sentiment predicts less well than do its components. Willingness to consume appears to be a complex construct, that is better captured by the inclusion of multiple indicators than by the inclusion of the Index created from those indicators.

The preceding consideration has focused on the consumer confidence to aggregate consumption (or its components) relationships. The literature provides us with relatively few analyses of the relationship between consumer confidence and other measures of household economic activity. Rouwendal and Longhi (2008) find a strong relationship between the development of house prices and the Dutch index of consumer confidence. Dawson and Henley (2012) investigate the association between unrealized financial expectations (over-optimism) and the subsequent mortgage repayment difficulties using British longitudinal data. Evidence is provided showing that over-optimism is associated with an increased likelihood of mortgage arrears. The results of Lamdin (2008) generally show that changes in the consumer sentiment measure are related to subsequent changes in revolving credit use. Brown et al. (2005) find empirical support for the hypothesis that optimistic financial expectations positively affect the amount of outstanding debt and growth in debt. Furthermore, Brown and Taylor (2006) suggest that financial optimism is inversely associated with saving and that the current financial expectations serve to predict future consumption.

Consumer Confidence Indexes can be sensibly used as social indicators in economic and social research (Zagórski and McDonnell 1995). According to Malgarini and Margani (2007), sentiment does not seem to be well explained by economic fundamentals alone because it also captures the effects of the political cycle and exceptional circumstances. The results of Starr (2012) are consistent with previous studies confirming that a substantial part of variation in consumer confidence is due to non-fundamentals. Bialowolski and Weziak-Bialowolska (2014) suggest that combining subjective and objective indicators enables one to capture the development of household financial situations differently. This approach seems to be both a natural solution for acquiring a broader picture and a more reliable basis for forecasts and policy assessments.

The idea of this study is to bring together the strengths of objective indicators (such as economic fundamentals) and subjective indicators (such as consumer confidence) and to make sense of the discrepancies that they show (as recommended by Veenhoven 2002) to improve household financial behavior forecasting. This study investigates the effects of consumer confidence (in the context of objective economic indicators) on not only household consumption/saving but also borrowing behavior, which are unsatisfactorily considered in previous discussions. In addition to aggregate confidence indicators, their components are used to provide more precise information.

Most studies examining the relationship between consumer confidence and household economic activity have focused on advanced economies. Studies of former socialist economies in Central and Eastern Europe are sparse. Because these countries are emerging market economies that have relatively less experience in dealing with financial crises, research on household financial behavior is particularly relevant. Some aspects of changes in household saving behavior in Poland were discussed by, among others Roszkiewicz (2006), Rytelewska and Kłopocka (2010), Debski and Swiderski (2011), Liberda and Pęczkowski (2012), Anioła and Gołaś (2013), Kośny and Piotrowska (2013), Roszkiewicz (2014), and Kolasa and Liberda (2015). This paper contributes to filling the gap in the literature by addressing the issue of household saving and borrowing behavior in the context of changing consumer confidence in Poland. The research described in this article is carried out on such a scale for the first time and is a continuation of research started by author previously.

The rest of the paper is organized as follows. In Sect. 2, basic statistics related to Polish credit market for households is revealed. Section 3 briefly describes the data and the methodology of the research. Section 4 presents and discusses the empirical findings of regression analysis. Section 5 concludes with some remarks.

## Polish Credit Market for Households

**Fig. 1:**
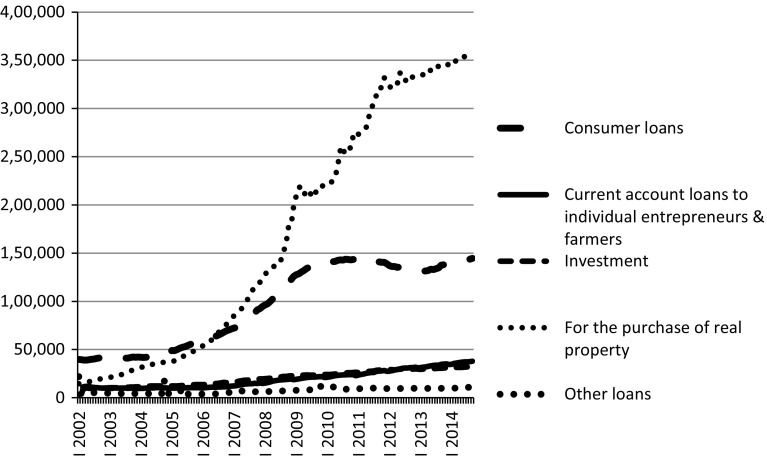
Monetary Financial Institutions loans to households—stocks in PLN million. *Source*: Narodowy Bank Polski data

The period under analysis (2002Q1–2014Q3) covers a time of substantial changes in the Polish credit market for households. Poland’s household debt has tripled relative to GDP as well as in terms of disposable income over the past decade and is now one of the highest in the Central Eastern and Southeastern Europe. It went up from about 20 % of disposable income in the early 2000s to 58 % in 2013 (IMF 2015). The most visible increase took place in the market for mortgages. Consumer credit was also subject to considerable growth between 2002 and 2009, but afterwards this growth stopped, and in the period 2009–2013 the penetration rate decreased (Fig. 1).

The development of mortgages was accompanied by changes in the housing market. The best indicator of housing prices, the average price of apartments in the 16 main cities, has stabilised since the middle of 2013 after a decline of about 30 % in real terms since its 2007Q1 peak. Thanks in part to tighter prudential regulations applying to mortgages, the bursting of the housing bubble led to a correction of about two-thirds of the rise recorded in the 2005–2007 boom. However, the impact of tumbling house prices has been contained owing to modest wealth effects, interest rate cuts and restrictions on borrowing by low-income households. As a result, although the share of non-performing mortgages has increased steadily, it remains limited (OECD 2014).

**Fig. 2:**
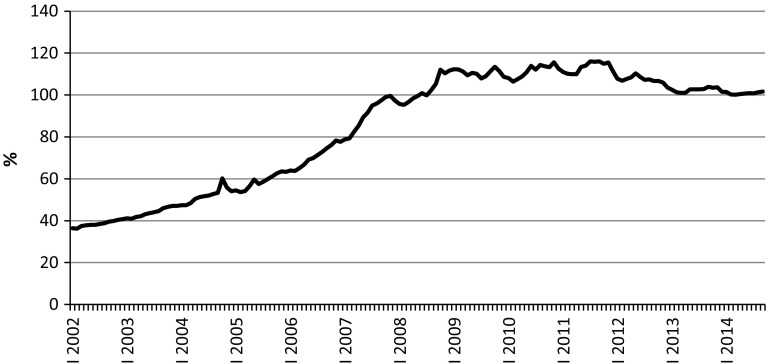
Household loans-to-deposits ratio. *Source*: own calculations based on Narodowy Bank Polski data

The loans-to-deposits ratio is much greater than before the boom, although it has stabilized since 2009 and even drifted down recently (Fig. 2). Growing indebtedness, allowing growth of consumption above this of incomes (and also enabling growth of housing sector) was underpinned by flows of external finance to the country, but also by the pressure of household needs previously suppressed (Lissowska 2015).

## Data and Methods

This research is based on a selection of indicators derived from national accounts that illustrate the behavior of households concerning the propensity to save and borrow.

Households’ saving is defined as the difference between their gross disposable income (mainly wages received, revenue of the self-employed and net property income) and their consumption (expenditure on goods and services). In other words, gross saving is the part of the gross disposable income which is not spent as final consumption expenditure. Saving rates can be considered either the gross or net of consumption of fixed capital. This analysis focuses on gross measures. The gross household saving rate is calculated by dividing gross saving by gross disposable income. Gross disposable income is usually adjusted for the change in net equity of households in pension fund reserves.

For the purposes of this study, two measures of household propensity to save are employed:

- *HSRtot—*the gross household saving rate, with gross disposable income being adjusted for the change in net equity of households in pension fund reserves, hereafter called total household saving rate
  1 $$HSRtot = \frac{DI + PF - C}{DI}$$
- *HSRvol*—the gross household saving rate, with gross disposable income not being adjusted for the change in net equity of households in pension fund reserves, hereafter called voluntary household saving rate
  2 $$HSRvol = \frac{DI - C}{DI}$$
  where *DI* is gross disposable income, *PF* is the change in net equity of households in pension fund reserves, *C* is consumption.

The first measure is widely used in international statistics. The second measure seems to be more appropriate in the context of this study. We assume that consumer confidence contributes to household consumption/saving decisions. As, in general terms, the change in net equity of households in pension fund reserves is not the subject of household decisions (the vast majority of the transfers to the pension funds are mandatory), including it in the household saving rate may lead to underestimation of consumer confidence to household saving relationship. Voluntary household saving rate reflects the unconstrained saving of households and may be more sensitive to consumer confidence than total household saving rate is. Higher values of household saving rates represent a higher household propensity to save.

The saving rates considered here are the “flow” measures and do not reflect variations in the “stock” of wealth of households. Holding gains or losses on assets and liabilities, particularly realized and unrealized gains/losses on equities or real estate, are not included in the national accounts measures of savings.

Savings is a source used by households to finance investment. If households have an excess of savings over investment that they can provide to other sectors of the national economy or to non-residents (e.g., by making bank deposits or buying shares), it means that they are net lenders. In contrast, households are net borrowers when they (considering the sector as a whole) need to borrow money from other sectors to finance their investment and other capital transactions. In this study, household propensity to borrow is measured by the household borrowing rate (hereafter *HBR*) calculated by dividing the net lending/borrowing of households by gross disposable income. A lower household borrowing rate value indicates that the household propensity to borrow is higher. All data used for *HSRtot*, *HSRvol* and *HBR* evaluation are derived from Polish quarterly national accounts compiled by the Central Statistical Office based on ESA 2010. Unadjusted quarterly data show large fluctuations. To smooth fluctuating series ratios based on four-quarter-cumulated sums (the value of three preceding quarters added to that of the quarter concerned) are used, e.g.,

3 $$HSRtot_{t} = \frac{{\mathop \sum \nolimits_{i = 0}^{3} DI_{t - i} + \mathop \sum \nolimits_{i = 0}^{3} PF_{t - i} - \mathop \sum \nolimits_{i = 0}^{3} C_{t - i} }}{{\mathop \sum \nolimits_{i = 0}^{3} DI_{t - i} }}$$

Concerning the confidence indicators, we use data calculated by the Central Statistical Office in cooperation with the National Bank of Poland. Two composite indexes of consumer confidence (current and forward consumer confidence indexes) and their components are adopted as independent variables describing consumer confidence. The two composite indexes of consumer confidence are as follows:

1. The Current Consumer Confidence Index (*CCCI*) indicates the sentiment of consumers based on their opinions on the financial condition of their own household, domestic economy, and conditions for making important purchases. The index takes values between −100 and 100; a positive value signifies that the majority of consumers have a good opinion of their own and the economy’s condition. However, a negative value suggests that a higher number of consumers hold an opposing view.
2. The Forward Consumer Confidence Index (*FCCI*) represents the predictions of consumers concerning changes in the financial condition of their households and the Polish economy in the next 12 months. The index takes values between −100 and 100; a positive value signifies that the majority of consumers are optimistic about changes that will occur in the next 12 months. However, a negative value indicates that a higher number of consumers hold a pessimistic view.

The values of composite indexes are calculated with the use of values of component indexes. The value of a component index is calculated by multiplying the percentage share of a given response to a question by its weight and adding up the products obtained for all responses to the question, e.g.,

4 $$I1 = \mathop \sum \limits_{i = 1}^{r} PS_{i} \times W_{i} ,$$

where *PS* is the percentage share of a given response to a question, *W* is the weight of a given response to a question, *r* is the number of the response options to a question.

The questions on which the two indexes, *CCCI* and *FCCI*, are based, together with the response options and their weights, are provided in Table 1.

**Table 1:** Questions from a questionnaire concerning consumer confidence in Poland used to estimate the *CCCI* and *FCCI.* *Source*: GUS (2004, pp. 21–24)

Symbol of the component index	Question	Response options	Weight
*I*1	How has the financial condition of your household changed in the last 12 months?	It is much better	1.0
		It is slightly better	0.5
		It has remained the same	0.0
		It is slightly worse	−0.5
		It is much worse	−1.0
		I do not know	0.0
*I*2	How do you expect the financial condition of your household will change in the next 12 months?	It will be much better	1.0
		It will be slightly better	0.5
		It will remain the same	0.0
		It will be slightly worse	−0.5
		It will be much worse	−1.0
		I do not know	0.0
*I*3	How do you evaluate the changes in the general condition of the economy in the last 12 months?	It is much better	1.0
		It is slightly better	0.5
		It has remained the same	0.0
		It is slightly worse	−0.5
		It is much worse	−1.0
		I do not know	0.0
*I*4	How do you expect the general condition of your country’s economy will change in the next 12 months?	It will be much better	1.0
		It will be slightly better	0.5
		It will remain the same	0.0
		It will be slightly worse	−0.5
		It will be much worse	−1.0
		I do not know	0.0
*I*7	How do you expect the level of unemployment in your country will change in the next 12 months?	It will increase considerably	−1.0
		It will slightly increase	−0.5
		It will remain the same	0.0
		It will slightly decrease	0.5
		It will decrease considerably	1.0
		I do not know	0.0
*I*8	Considering the general condition of the country’s economy, do you think now is the right time for people to make major purchases?	Yes, now is the right time	1.0
		It is neither the right nor a bad time	0.5
		No, it is not the right time	−1.0
		I do not know	0.0
*I*11	How likely do you think it is that in the next 12 months you will save any sum of money?	Very likely	1.0
		Quite likely	0.5
		Unlikely	−0.5
		Absolutely unlikely	−1.0
		I do not know	0.0

The mathematical equation of the *CCCI* is as follows:

5 $$CCCI = \frac{I1 + I2 + I3 + I4 + I8}{5},$$

where *I*1, *I*2, *I*3, *I*4, *I*8 are empirical values of component indexes from Table 1. The mathematical equation of the *FCCI* is as follows:

6 $$FCCI = \frac{I2 + I4 + I7 + I11}{4},$$

where *I*2, *I*4, *I*7, *I*11 are empirical values of component indexes from Table 1.

The explanatory variables that we treat as “economic fundamentals” are variables that are usually found to have some predictive power to explain changes in consumption. They include the following:

- the real gross household disposable income in PLN billions (current values are deflated by consumer price index) (hereafter *DIr*), published by the Central Statistical Office,
- the average Monetary Financial Institutions interest rate on outstanding amounts of deposits in PLN with agreed maturity of households and non-profit institutions serving households (hereafter *IRD*), published by the National Bank of Poland,
- the average Monetary Financial Institutions interest rate on outstanding amounts of loans in PLN (overdraft excluded) of households and non-profit institutions serving households (hereafter *IRL*), published by the National Bank of Poland.

**Fig. 3:**
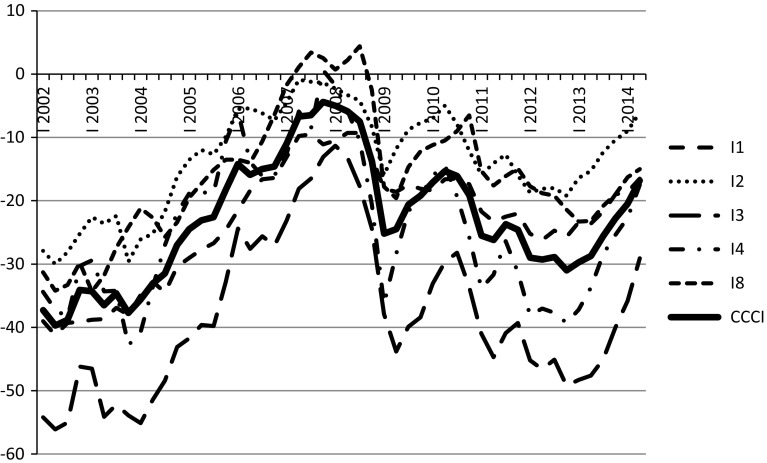
Current Consumer Confidence Index (*CCCI*) and its underlying indices. *Source*: GUS data

**Fig. 4:**
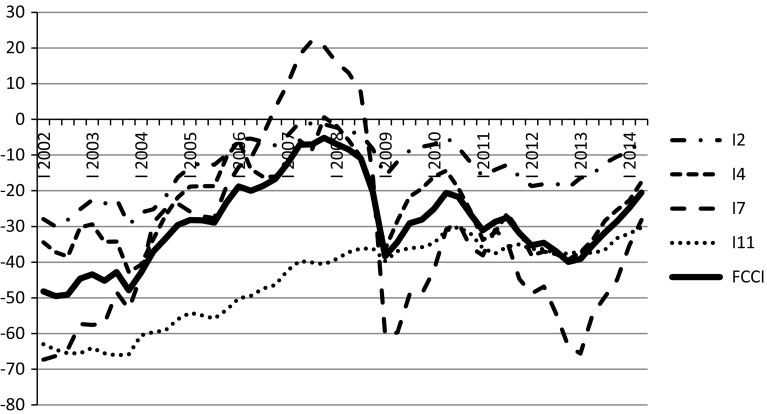
Forward Consumer Confidence Index (*FCCI*) and its underlying indices. *Source*: GUS data

The dataset used covers quarterly observations in the period from 2002Q1 to 2014Q3. The period under analysis is determined by the availability of data. Figures 3 and 4 show Current Consumer Confidence Index and Forward Consumer Confidence Index with their underlying indices, respectively.

The questions of interest are first, whether indexes of consumer confidence have any predictive power on their own for future household saving and borrowing rates, and second, whether they contain information about future household saving and borrowing rates aside from the information contained in other available indicators. Multiple linear regression analysis (OLS technique) is used to answer these questions. Initially, Augmented Dickey–Fuller tests are performed in order to determine the order of integration of the variables. Most variables are found to be integrated of order one or I(1) [interest rate on outstanding amounts of loans is the exception as it is I(0)]. Therefore all variables are first-differenced and changes in household saving and borrowing rates are modeled as functions of changes in other economic variables. Thus, no variable in level enter household saving and borrowing models. The descriptive statistics of variables are presented in Table 2.

**Table 2:** Descriptive statistics

Variables	Min	Max	Mean	SD
*Levels*				
Total household saving rate (*HSRtot*) (percentage)	1.68	11.58	5.01	2.75
Voluntary household saving rate (*HSRvol*) (percentage)	−0.72	9.56	2.43	2.91
Household borrowing rate (*HBR*) (percentage)	−5.52	5.29	−2.11	2.94
Past financial situation (*I*1) (points)	−41.20	−9.30	−23.31	9.25
Expected financial situation (*I*2) (points)	−30.00	−0.90	−13.51	8.16
Past general economic situation (*I*3) (points)	−56.10	−11.30	−37.89	12.39
Expected general economic situation (*I*4) (points)	−42.80	0.60	−24.19	11.39
Major purchases (*I*8) (points)	−34.30	4.40	−15.98	10.15
Current Consumer Confidence Index (*CCCI*) (points)	−39.70	−4.40	−22.97	9.72
Unemployment (*I*7) (points)	−67.30	22.00	−32.45	25.62
Saving plans (*I*11) (points)	−66.10	−30.20	−45.07	11.93
Forward Consumer Confidence Index (*FCCI*) (points)	−49.50	−5.20	−28.89	12.03
Deposit interest rate (*IRD*) (percentage)	2.43	7.16	3.90	1.11
Loan interest rate (*IRL*) (percentage)	6.97	19.00	10.49	2.41
Disposable income (*DIr*) (PLN billions)	136.01	258.69	190.46	38.67
*First differences*				
∆Total household saving rate (∆*HSRtot*) (percentage points)	−1.69	1.42	−0.20	0.70
∆Voluntary household saving rate (∆*HSRvol*) (percentage points)	−1.74	1.33	−0.19	0.73
∆Household borrowing rate (∆*HBR*) (percentage points)	−2.65	1.85	−0.23	0.90
∆Past financial situation (∆*I*1) (points)	−5.00	4.10	0.46	2.03
∆Expected financial situation (∆*I*2) (points)	−7.60	5.50	0.45	2.85
∆Past general economic situation (∆*I*3) (points)	−13.50	8.80	0.51	4.56
∆Expected general economic situation (∆*I*4) (points)	−15.70	9.20	0.35	5.50
∆Major purchases (*I*8) ∆ (points)	−15.30	4.90	0.33	3.67
∆Current Consumer Confidence Index (∆*CCCI*) (points)	−11.40	4.70	0.42	3.15
∆Unemployment (∆*I*7) (points)	−44.50	14.80	0.80	9.58
∆Saving plans (∆*I*11) (points)	−4.10	5.30	0.67	1.85
∆Forward Consumer Confidence Index (∆*FCCI*) (points)	−17.90	5.90	0.57	4.34
∆Deposit interest rate (∆*IRD*) (percentage points)	−1.12	1.20	−0.09	0.43
∆Loan interest rate (∆*IRL*) (percentage points)	−2.33	0.45	−0.24	0.49
∆Disposable Income (∆*DIr*) (PLN billions)	−1.17	5.69	2.46	1.64

NBP average exchange rate of 1 USD in PLN of 2016.05.02 is 3.8195

Two questions of interest require the two-step process that is explained upon the example of total household saving rate. The same method is applied for voluntary household saving rate and household borrowing rate.

The first step of analysis is dedicated to answer the question whether changes in indexes of consumer confidence have any predictive power on their own. The first difference of total household saving rate is regressed against four lags of the first difference of the given consumer confidence index as the explanatory variable. The procedure is realized for each of nine consumer confidence indexes separately. This specification takes the following form:

7 $$\Delta HSRtot_{t} =\, \propto_{0} + \mathop \sum \limits_{i = 1}^{4} \beta_{i}\Delta I_{t - i} + \varepsilon_{t}$$

where ∆*I*_*t*−*i*_ {*i* = 1,…,4} are lagged values of the change in given consumer confidence index, $\varepsilon_{t}$ is the error term. Moreover, the first difference of total household saving rate is regressed against first differences of all component confidence indicators of one composite confidence index, for each of lags *i*, independently:

8 $$\Delta HSRtot_{t} =\, \propto_{0} + \mathop \sum \limits_{n} \vartheta_{n}\Delta In_{t - i} + \varepsilon_{t} ,$$

where n = 1, 2, 3, 4, 8 for CCCI or n = 2, 4, 7, 11 for FCCI.

The second step of regression analysis involves investigating whether consumer confidence has any predictive ability once controls for information contained in other variables are introduced. This is done by calculating a baseline model in which the change in total household saving rate depends only on lagged changes in fundamental variables

9 $$\Delta HSRtot_{t} =\, \propto_{0} + \mathop \sum \limits_{{m \in \left\{ {1,2,3,4} \right\}}} \mathop \sum \limits_{j = 1}^{2} \gamma_{jm}\Delta Z_{jt - m} + \varepsilon_{t}$$

and a model that modifies Eq. (9) by introducing lagged change in the given consumer confidence index (alternately, each of four lags *i*), producing the following form,

10 $$\Delta HSRtot_{t} =\, \propto_{0} + \mathop \sum \limits_{{m \in \left\{ {1,2,3,4} \right\}}} \mathop \sum \limits_{j = 1}^{2} \gamma_{jm}\Delta Z_{jt - m} + \beta\Delta I_{t - i} + \varepsilon_{t} ,$$

where ∆*Z*_*jt*−*m*_ is lag *m* of the change in fundamental variable *Z*_*j*_. Then the baseline model (Eq. 9) is compared with an alternative that includes both lagged changes in fundamental variables and lagged change in the given consumer confidence index (Eq. 10). A significant change in the $\bar{R}^{2}$ statistic (using an *F* test to determine significance) is interpreted as an indication that the newly added variable (lag *i* of the change in customer confidence index) offers significant additional predictive power for the dependent variable (change in total household saving rate) over variables previously included in the regression model (lagged changes in economic fundamentals). Moreover, AIC values of the baseline model (Eq. 9) and its alternatives (following Eq. 10) are compared. To enhance the assessment of consumer confidence predictive power, models with lagged dependent variables are also considered, as follows

11 $$\Delta HSRtot_{t} =\, \propto_{0} + \mathop \sum \limits_{{m \in \left\{ {1,2,3,4} \right\}}} \mathop \sum \limits_{j = 1}^{2} \gamma_{jm}\Delta Z_{jt - m} + \rho\Delta HSRtot_{t - s} + \varepsilon_{t} ,$$

12 $$\Delta HSRtot_{t} =\, \propto_{0} + \mathop \sum \limits_{{m \in \left\{ {1,2,3,4} \right\}}} \mathop \sum \limits_{j = 1}^{2} \gamma_{jm}\Delta Z_{jt - m} + \rho\Delta HSRtot_{t - s} +\Delta I_{t - i} + \varepsilon_{t} ,$$

where ∆*HSRtot*_*t*−*s*_ is lag *s* of the change in total household saving rate.

The choice of which fundamental variables to include in the regression is inherently somewhat arbitrary. After a preliminary analysis of a broader set of fundamentals, three fundamental variables described above (changes in real gross household disposable income, interest rate on outstanding amounts of deposits, and interest rate on outstanding amounts of loans) are chosen. Changes in household saving rates are regressed against changes in: disposable income and interest rate on outstanding amounts of deposits, change in household borrowing rate is regressed against changes in disposable income and interest rate on outstanding amounts of loans. The number of variables is to be limited to a necessary minimum given that a sample consists of only 47 observations (51 minus 3 due to the four-quarter-cumulated sums nature of saving and borrowing rates, minus 1 due to first-differences). For the same reason models with only one lag of each variable are preferred. The decision which of four lags to use is made based on the evidence provided by Akaike’s Information Criterion (AIC).

Both the research questions and methodology applied in this study are inspired by these of Carroll et al. (1994). However, they study the predictive power of consumer sentiment on household spending, whereas the predictive power of consumer sentiment on household saving and borrowing rates is examined in the present paper. The left-hand side variable in their regressions is the log difference of the indicated category of real household spending while changes in the smoothed saving or borrowing rates are regressed here. Household saving rate seems to be most commonly used measure of household propensity to save. Analogous measure for borrowing behavior is applied. Moreover, Carroll et al. (1994) use only one composite sentiment index (the University of Michigan’s Index of Consumer Sentiment) while in this study in addition to aggregate confidence indicators, their components are used to provide more precise information. There are also some differences in the set of fundamentals. In particular, changes in interest rates on outstanding amounts of deposits/loans are included here.

## Empirical Results

The following section presents and discusses empirical findings of a regression analysis. For better understanding it is worth reminding that the left-hand-side variable is the first difference in a smoothed version of the given ratio.

Table 3 summarizes the appraisal of the predictive ability of models with only four lags of changes in consumer confidence indexes as explanatory variables (according to Eq. 7). Results demonstrate a statistically significant relationship between changes in analyzed ratios and lagged changes in some consumer confidence indexes.

**Table 3:** Forecast of Changes in Household Saving and Borrowing Rates with Four Lags of Changes in Consumer Confidence Indicators

		∆*HSRtot*		∆*HSRvol*		∆*HBR*	
		$\bar{R}^{2}$	AIC	$\bar{R}^{2}$	AIC	$\bar{R}^{2}$	AIC
1	∆*I*1	−0.052	106.33	−0.031	108.99	−0.04	127.94
	Past Financial Situation	(0.823)		(0.679)		(0.792)	
2	∆*I*2	−0.042	105.9	−0.046	109.69	−0.025	127.31
	Expected Financial Situation	(0.474)		(0.562)		(0.235)	
3	∆*I*3	0.019	103.14	0.027	106.36	0.007	125.84
	Past general economic situation	(0.344)		(0.277)		(0.591)	
4	∆*I*4	0.027	102.72	0.028	106.3	0.008	125.81
	Expected general economic situation	(0.218)		(0.265)		(0.272)	
5	∆*I*8	0.063*	101.02	0.082**	103.68	0.194**	116.24
	Present Major Purchases Climate	(0.078)		(0.025)		(0.018)	
6	∆*CCCI*	0.026	102.81	0.034	106.03	0.048	123.92
	Current Consumer Confidence Index	(0.245)		(0.164)		(0.214)	
7	∆*I*7	0.232***	91.88	0.217***	96.33	0.300***	109.76
	Expected Unemployment Level	(0.000)		(0.000)		(0.000)	
8	∆*I*11	−0.087	107.82	−0.059	110.23	−0.067	129.13
	Future Savings Likelihood	(0.983)		(0.729)		(0.738)	
9	∆*FCCI*	0.112***	98.55	0.117***	101.87	0.150***	118.7
	Forward Consumer Confidence Index	(0.002)		(0.000)		(0.001)	

The table reports regressions according to Eq. 7. The numbers in parentheses are *p* values of the joint significance of four lags of change in the given customer confidence index. The number of observations (N) is 46. Hypothesis tests were conducted using a heteroskedasticity and serial correlation robust covariance matrix. *HSRtot*, *HSRvol*, *HBR* denote total household saving rate, voluntary household saving rate, household borrowing rate, respectively

* Statistical significance at the 10 % level; ** statistical significance at the 5 % level; *** statistical significance at the 1 % level

The highest influence is exerted by lagged change in index *I*7, which addresses household expectation concerning unemployment level. Lagged values of change in *I*7, taken on their own, explain about 23, 22, and 30 % of the variation in changes in total household saving rate, voluntary household saving rate and household borrowing rate, respectively. The probability that this explanatory power was generated merely by chance is estimated to be essentially nil (row 7, number in parentheses). Lagged values of change in index *I*8 (row 5), which gives the present purchasing climate appraisal explain approximately 6, 8, 19 % of the variation in changes in total household saving rate, voluntary household saving rate and household borrowing rate, respectively. Four lags of changes in each of remaining component indexes (*I*1 and *I*2, that show the evaluation of household financial situation, *I*3 and *I*4, relating to the general economic situation, and *I*11, reflecting the likelihood of household future savings) are not jointly significant at any of the usual levels. With regard to composite indexes, this is Forward Consumer Confidence Index (row 9), lagged changes of which explain about 11, 12, and 15 % of the variation in changes in total household saving rate, voluntary household saving rate and household borrowing rate, respectively. In each of these regressions, the coefficients on four lags of changes in *FCCI* are jointly significant at better than the 1-percent level.

Table 4 reveals regressions following Eq. 8. This specification enables to compare the effects exerted by lagged changes in components of Current versus Forward Consumer Confidence Indexes for different time lags. Interestingly, changes in household saving and borrowing rates are better predicted by lagged changes in components of Forward than of Current Consumer Confidence Index. It is worth noting that the uppermost goodness of fit of the models is reached when third lags of explanatory variables are used. The evidence from both Tables 3 and 4 suggest that the best forecasting power is found for models of changes in household borrowing rate.

**Table 4:** Forecast of changes in household saving and borrowing rates with one lag of changes in component consumer confidence indicators

		Lag	∆*HSRtot*		∆*HSRvol*		∆*HBR*	
			$\bar{R}^{2}$	AIC	$\bar{R}^{2}$	AIC	$\bar{R}^{2}$	AIC
1	∆*I*1 + ∆*I*2 + ∆*I*3 + ∆*I*4 + ∆*I*8	*t* − 1	−0.079	109.58	−0.067	112.74	−0.063	131.69
	Components of Current Consumer Confidence Index		(0.316)		(0.293)		(0.400)	
2	∆*I*1 + ∆*I*2 + ∆*I*3 + ∆*I*4 + ∆*I*8	*t* − 2	−0.001	106.09	−0.016	110.43	0.029	127.46
	Components of Current Consumer Confidence Index		(0.151)		(0.222)		(0.205)	
3	∆*I*1 + ∆*I*2 + ∆*I*3 + ∆*I*4 + ∆*I*8	*t* − 3	0.056	103.29	0.077	105.91	0.107***	123.54
	Components of Current Consumer Confidence Index		(0.250)		(0.103)		(0.009)	
4	∆*I*1 + ∆*I*2 + ∆*I*3 + ∆*I*4 + ∆*I*8	*t* − 4	−0.053	107.22	−0.051	110.74	−0.038	128.73
	Components of Current Consumer Confidence Index		(0.320)		(0.279)		(0.219)	
5	∆*I*2 + ∆*I*4 + ∆*I*7 + ∆*I*11	*t* − 1	−0.049	107.4	−0.039	110.63	−0.031	129.39
	Components of Forward Consumer Confidence Index		(0.541)		(0.463)		(0.494)	
6	∆*I*2 + ∆*I*4 + ∆*I*7 + ∆*I*11	*t* − 2	0.008	104.8	−0.010	109.31	0.090**	123.54
	Components of Forward Consumer Confidence Index		(0.135)		(0.148)		(0.029)	
7	∆*I*2 + ∆*I*4 + ∆*I*7 + ∆*I*11	*t* − 3	0.318***	87.2	0.277***	93.56	0.349***	107.80
	Components of Forward Consumer Confidence Index		(0.000)		(0.000)		(0.000)	
8	∆*I*2 + ∆*I*4 + ∆*I*7 + ∆*I*11	*t* − 4	0.055*	101.4	0.058**	104.84	0.040**	124.30
	Components of Forward Consumer Confidence Index		(0.066)		(0.031)		(0.015)	

The table reports regressions according to Eq. 8. The numbers in parentheses are *p* values of the joint significance of one lag (specified in column 1) of changes in the component customer confidence indexes. The number of observations (N) is 47 for lags 1–3, and 46 for lag 4. Hypothesis tests were conducted using a heteroskedasticity and serial correlation robust covariance matrix. *HSRtot*, *HSRvol*, *HBR* denote total household saving rate, voluntary household saving rate, household borrowing rate, respectively

* Statistical significance at the 10 % level; ** statistical significance at the 5 % level; *** statistical significance at the 1 % level

The results of a regression analysis with the set of fundamental variables are demonstrated in Tables 5, 6. In Table 5 the baseline models regress the first differences of saving and borrowing rates against just lagged changes in fundamentals (according to Eq. 9), while in Table 6 both lagged changes in fundamentals and lagged dependent variables are used as explanatory variables (according to Eq. 11). As mentioned earlier, models with only one lag of each variable are preferred. Akaike’s Information Criterion (AIC) is used to choose between alternative models with different lag order. The minimum AIC is found when change in total/voluntary household saving rate is regressed against third lag of change in real gross household disposable income and fourth lag of change in interest rate on outstanding amounts of deposits. In case of the first-differenced household borrowing rate regression, there are two (first and fourth) lags of change in interest rate on outstanding amounts of loans taken (apart from third lag of change in real gross household disposable income). The reason is to improve the forecasting accuracy of the baseline regression as no model with just one of four lags of change in interest rate and one of four lags of change in disposable income produces the $\bar{R}^{2}$ statistic above 0.05. Header rows of Tables 5, 6 provide information on variables as well as values of the $\bar{R}^{2}$ and AIC statistics of the baseline models.

**Table 5:** Forecast of changes in household saving and borrowing rates, augmented by one lag of changes in consumer confidence indicators (baseline models without lagged dependent variable)

		∆*HSRtot*			∆*HSRvol*			∆*HBR*		
		Baseline model regressors ∆*DIr*_*t*−3_, ∆*IRD*_*t*−4_			Baseline model regressors ∆*DIr*_*t*−3_, ∆*IRD*_*t*−4_			Baseline model regressors ∆*DIr*_*t*−3_, ∆*IRL*_*t*−1_, ∆*IRL*_*t*−4_		
		$\bar{R}^{2}$ = 0.155, AIC = 94.47			$\bar{R}^{2}$ = 0.110, AIC = 100.40			$\bar{R}^{2}$ = 0.171, AIC = 116.65		
		Incremental $\bar{R}^{2}$	AIC	Lag	Incremental $\bar{R}^{2}$	AIC	Lag	Incremental $\bar{R}^{2}$	AIC	Lag
1	∆*I*1	−0.013	96.06	*t* − 1	−0.010	101.85	*t* − 3	−0.011	118.14	*t* − 3
	Past Financial Situation	(0.473)			(0.409)			(0.488)		
2	∆*I*2	−0.001	95.43	*t* − 4	−0.011	101.89	*t* − 3	0.005	117.27	*t* − 3
	Expected Financial Situation	(0.231)			(0.329)			(0.178)		
3	∆*I*3	−0.006	95.71	*t* − 1	0.008	100.94	*t* − 3	0.036	115.47^a^	*t* − 3
	Past general economic situation	(0.367)			(0.222)			(0.145)		
4	∆*I*4	0.018	94.35^a^	*t* − 3	0.032*	99.66^a^	*t* − 3	0.034*	115.58^a^	*t* − 3
	Expected general economic situation	(0.152)			(0.084)			(0.097)		
5	∆*I*8	0.002	95.25	*t* − 3	0.024	100.07^a^	*t* − 3	0.094***	112.02^a^	*t* − 3
	Present Major Purchases Climate	(0.360)			(0.163)			(0.008)		
6	∆*CCCI*	0.004	95.14	*t* − 3	0.025	100.02^a^	*t* − 3	0.054*	114.44^a^	*t* − 3
	Current Consumer Confidence Index	(0.280)			(0.102)			(0.058)		
7	∆*I*7	0.122***	88.18^a^	*t* − 3	0.144***	93.21^a^	*t* − 3	0.155***	108.04^a^	*t* − 3
	Expected Unemployment Level	(0.000)			(0.000)			(0.000)		
8	∆*I*11	−0.013	96.08	*t* − 4	0.006	101.04	*t* − 4	−0.007	117.94	*t* − 4
	Future Savings Likelihood	(0.452)			(0.176)			(0.268)		
9	∆*FCCI*	0.055**	92.24^a^	*t* − 3	0.077***	97.17	*t* − 3	0.085***	112.54^a^	*t* − 3
	Forward Consumer Confidence Index	(0.015)			(0.003)			(0.005)		

The table reports regressions according to Eqs. 9–10. The numbers in parentheses are *p* values of the significance of one lag (specified in column 3, 6 or 9) of change in the customer confidence index. The decision which of four lags of change in confidence index to choose is based on the AIC. The number of observations (N) is 46. Hypothesis tests were conducted using a heteroskedasticity and serial correlation robust covariance matrix. *HSRtot*, *HSRvol*, *HBR* denote total household saving rate, voluntary household saving rate, household borrowing rate, respectively. *DIr* indicates the real gross household disposable income, *IRD* and *IRL* signify interest rate on deposits and loans, respectively

* Statistical significance at the 10 % level; ** statistical significance at the 5 % level; *** statistical significance at the 1 % level

^a^Improvement in the AIC

**Table 6:** Forecast of changes in household saving and borrowing rates, augmented by one lag of changes in consumer confidence indicators (baseline models with lagged dependent variable)

		∆*HSRtot*			∆*HSRvol*			∆*HBR*		
		Baseline model regressors ∆*DIr*_*t*−3_, ∆*IRD*_*t*−4_, ∆*HSRtot*_*t*−4_			Baseline model regressors ∆*DIr*_*t*−3_, ∆*IRD*_*t*−4_, ∆*HSRvol*_*t*−4_			Baseline model regressors ∆*DIr*_*t*−3_, ∆*IRL*_*t*−1_, ∆*IRL*_*t*−4_, ∆*HBR*_*t*−3_		
		$\bar{R}^{2}$ = 0.236, AIC = 83.04			$\bar{R}^{2}$ = 0.178, AIC = 89.95			$\bar{R}^{2}$ = 0.319, AIC = 103.05		
		Incremental $\bar{R}^{2}$	AIC	Lag	Incremental $\bar{R}^{2}$	AIC	Lag	Incremental $\bar{R}^{2}$	AIC	Lag
1	∆*I*1	−0.013	84.63	*t* − 1	−0.016	91.66	*t* − 3	−0.008	104.40	*t* − 3
	Past Financial Situation	(0.324)			(0.573)			(0.463)		
2	∆*I*2	0.015*	83.08	*t* − 4	−0.011	91.42	*t* − 3	−0.001	103.93	*t* − 3
	Expected Financial Situation	(0.094)			(0.363)			(0.278)		
3	∆*I*3	−0.008	84.35	*t* − 1	−0.013	91.51	*t* − 2	0.006	103.52	*t* − 3
	Past General Economic Situation	(0.416)			(0.390)			(0.306)		
4	∆*I*4	−0.003	84.09	*t* − 3	0.008	90.45	*t* − 3	0.014	102.93^a^	*t* − 3
	Expected general economic situation	(0.329)			(0.196)			(0.193)		
5	∆*I*8	−0.015	84.78	*t* − 2	−0.017	91.73	*t* − 3	0.074***	98.82^a^	*t* − 3
	Present Major Purchases Climate	(0.607)			(0.642)			(0.009)		
6	∆*CCCI*	−0.008	84.38	*t* − 4	−0.005	91.13	*t* − 3	0.030	101.92^a^	*t* − 3
	Current Consumer Confidence Index	(0.287)			(0.331)			(0.103)		
7	∆*I*7	0.069***	79.87^a^	*t* − 3	0.092***	85.75^a^	*t* − 3	0.107***	96.38^a^	*t* − 3
	Expected Unemployment Level	(0.006)			(0.002)			(0.000)		
8	∆*I*11	0.011*	83.27	*t* − 2	0.000	90.82	*t* − 4	−0.008	104.36	*t* − 1
	Future Savings Likelihood	(0.096)			(0.264)			(0.439)		
9	∆*FCCI*	0.022*	82.67	*t* − 3	0.041**	88.62^a^	*t* − 3	0.055**	100.16^a^	*t* − 3
	Forward Consumer Confidence Index	(0.078)			(0.021)			(0.018)		

The table reports regressions according to Eqs. 11–12. The numbers in parentheses are *p* values of the significance of one lag (specified in column 3, 6 or 9) of change in the customer confidence index. The decision which of four lags of change in confidence index to choose is based on the AIC. The number of observations (N) is 43 for household saving models, and 44 for household borrowing models. Hypothesis tests were conducted using a heteroskedasticity and serial correlation robust covariance matrix. *HSRtot*, *HSRvol*, *HBR* denote total household saving rate, voluntary household saving rate, household borrowing rate, respectively. *DIr* indicates the real gross household disposable income, *IRD* and *IRL* signify interest rate on deposits and loans, respectively

* Statistical significance at the 10 % level; ** statistical significance at the 5 % level; *** statistical significance at the 1 % level

^a^Improvement in the AIC

In columns 1, 4, and 7, the upper entry in each cell reports the increment to the $\bar{R}^{2}$ provided by the lagged change in consumer confidence, while the lower entry (in parentheses) displays the *p* value from the test of the hypothesis that the coefficient of the lagged change in consumer confidence index equals zero. In columns 2, 5, and 8, the AIC statistics are recorded. The decision which of four alternative models with lagged change in the given consumer confidence index should be presented was made upon Akaike’s Information Criterion (AIC). Columns 3, 6, and 9 present which lag of change in consumer confidence index minimizes the AIC, and thus which model is exhibited.

As far as change in total household saving rate is considered, expanding the set of lagged changes in fundamental variables with lagged change in consumer confidence indicator yields a statistically significant positive effect in case of *I*7 (which demonstrates households expectations of the unemployment level) and Forward Consumer Confidence Index. The maximum improvement of the predictive ability is 12 % points with reference to the third lag in change in consumer confidence index *I*7 (Table 5 row 7 column 1). As a result, almost 28 % of the variation of change in the total household saving rate is explained. The enhancement in AIC is obtained by including lagged changes in *I*7, *FCCI* or *I*4 (that reveals expectations on general economic situation).

Adding the lagged value of change in total household saving rate to the baseline equation results in a remarkable increase in the predictive power of the regression (by 8.1 % points). Still, there is statistically significant raise of forecast accuracy from the lagged changes in customer confidence indicators in case of *I*2 (expected financial situation), *I*7 (expected unemployment level), *I*11 (likelihood of household future savings), and Forward Consumer Confidence Index (Table 6, column 1). It supports the earlier comment that lagged changes in future-oriented confidence indicators are better predictors of changes in household saving rate than lagged changes in indicators which evaluate past situation. The highest increase in predictive power, by approximately 7 % points with statistical significance at the 1-percent level, is recorded when the third lag of change in *I*7 is added. The other improvements are statistically significant at the 10-percent level.

Generally, similar pattern of results holds for changes in voluntary household saving rate. Again, this is the third lag of change in *I*7 that gives the best improvement in forecast accuracy (Table 5, column 4, row 7 and Table 6, column 4, row 7). An increase in the predictive power of the model at better than the 5-percent level is also gained by introducing third lag of change in *FCCI* (Table 5, column 4, row 9 and Table 6, column 4, row 9). The incremental $\bar{R}^{2}$ statistics are noteworthy higher for changes in voluntary than total household saving rate but $\bar{R}^{2}$ statistics of the baseline models (following Eqs. 9 and 11) are lower for changes in voluntary than total household saving rate (0.110 versus 0.155 for models without lagged explained variables and 0.178 versus 0.236 for models with lagged explained variables). Therefore, greater sensitivity of changes in voluntary household saving rate to changes in consumer confidence than that of changes in total household saving rate (adjusted for the change in net equity of households in pension fund reserves that, in general, is not the subject of household decisions) does not provide the unequivocal support for the hypothesis that if total household saving rate (as the measure of household propensity to save) is considered, the consumer confidence to household saving relationship is underestimated.

Columns 7–9 in Table 5 reveal results of implementing lagged changes in consumer confidence to the baseline regression of first-differenced household borrowing rate. Statistically significant increment in $\bar{R}^{2}$’s is gained in case of five out of nine indexes. AIC statistics are improved in six instances. Predictive power of the model is increased significantly at the 1-percent level by lagged changes in *I*8, *I*7, and *FCCI*. Third lag of change in *I*7 boosts $\bar{R}^{2}$ by almost 16 % points. In consequence, roughly 33 % of the variation of change in household borrowing rate is explained. Third lag of change in *I*8, which gives the contemporary purchasing climate appraisal, augments $\bar{R}^{2}$ by approximately 9 % points to the level of 26.5 %.

When lagged regressand is included as the right-hand-side variable (according to Eqs. 11–12), lagged changes in *I*8 and *I*7 retain their ability to significantly improve forecast accuracy at the 1-percent level (Table 6, column 7, rows 5 and 7, respectively). Lagged change in *FCCI* holds its predictive power at better than the 5-percent level (Table 6, column 7, row 9). Lagged changes in five out of nine consumer confidence indicators give the improvement in AIC statistics (column 8). These are third lags of changes in sentiments that provide the minimum AIC models in all but one cases (column 9).

Comparing three household financial behavior measures under considerations, it is worth emphasizing that the best predictive power of regressions is found for the change in household borrowing rate. These are household borrowing rate models, that are characterized by the highest prediction accuracy among just customer confidence models (Tables 3, 4). Moreover, expanding the set of lagged changes in fundamental variables with lagged changes in consumer confidence indicators yields the biggest effect in increment to $\bar{R}^{2}$'s of borrowing regressions (Table 5). This holds when lagged predicted variables are included in models as regressors (Table 6).

Generally, our results are in line with the broad body of the literature that stresses the importance of consumer confidence for stimulating household economic behavior. One of the strands in this literature confirms the usefulness of consumer confidence indicators as explanatory variables in household consumption forecasts (e.g., Carroll et al. 1994). One can expect that consumer confidence indicators should also improve household saving and borrowing forecasts.

In fact, our results provide convincing support for the premise that a part of variation in household saving and borrowing behavior is due to consumer confidence. Moreover, it has been demonstrated that some confidence indexes (subjective indicators) contain predictive ability beyond economic fundamentals (objective indicators). These results are consistent with earlier recommendations to combine subjective and objective indicators to achieve a broader picture and a more reliable basis for forecasts and policy assessments (Veenhoven 2002; Bialowolski and Weziak-Bialowolska 2014). Roszkiewicz (2014) also confirms the important role of subjective determinants of the accumulation of reserves.

To the best of our knowledge, this paper provides a unique appraisal of the predictive ability of not only composite but also component consumer confidence indexes for household saving and borrowing rates. Kellstedt et al. (2015) advise practitioners against the uncritical use of the ICS as a composite measure in their analyses, and prescribe instead that analysts consider using some subset of the component indicators, depending on the theoretical question at hand. Indeed, in our study some survey questions have more predictive power than composite indexes and other component questions.

The overwhelming forecasting ability is found for the question that ask about expected unemployment level. Similarly, Bram and Ludvigson (1998) discover that questions asking specifically about job prospects in the respondent’s area have the most explanatory power for consumer expenditures. One possible interpretation is that, as many households build their economic security solely on job stability (Kośny and Piotrowska 2013), a growth in uncertainty associated with job prospects triggers precautionary savings and substantially decreases households propensity to borrow. The second most influential component indicator that exhibits significant forecasting ability, especially for borrowing behavior, is the question referring to present climate for major purchases. Yet Bram and Ludvigson (1998) note that question about current buying conditions elicits virtually no incremental information for consumer spending. Surprisingly, lagged change in component index that directly relates to household saving prospects has practically no explanatory power for changes in household saving (and borrowing) rates.

Future changes in household saving and borrowing rates are better predicted by changes in components of Forward than of Current Consumer Confidence Index. This is in line with Ludvigson (2004) who investigates the consumer spending—consumer confidence relation and finds that the expectations component of both the Conference Board and Michigan overall confidence index exhibits more predictive power than the composite index. In the current study, measures of consumer confidence seem particularly useful at the longer, 3-quarter-ahead horizon. Wilcox (2007), likewise, shows that the individual component questions, and the aggregated ICS itself, provide much more reliable improvements in 4-quarter-ahead forecasts than they do for 1-quarter-ahead forecasts of consumption.

For a better understanding of our findings, an aspect of household saving and borrowing behavior complexity needs to be emphasized. Household financial behavior is a multifaceted phenomenon that reflects the influence of many factors of different natures. A topic deserving additional research attention is the motivation of saving. It seems worth examining further whether the relationship of consumer confidence to household saving is significant, irrespective of saving motives, or due to the circumstances of precautionary motive priority, as observed in Poland. The most likely high influence of the precautionary motive on household saving behavior in Poland is suggested, among others, by Kośny (2013).

A micro data analysis to clarify whether households with different saving motives reveal different sensitivity of saving behavior to consumer confidence is clearly out of the scope of this paper but is left for future research.

## Conclusions

This paper provides a unique appraisal of the predictive ability of not only composite but also component consumer confidence indexes for household saving and borrowing rates. The questions of interest are first, whether indexes of consumer confidence have any predictive power on their own for future household saving and borrowing rates, and second, whether they contain information about future household saving and borrowing rates aside from the information contained in other available indicators.

In general, the multiple linear regression analysis (OLS technique) of Polish time-series data gives positive answers to both questions. To be more specific, when changes in saving and borrowing rates are regressed against just lagged values of changes in confidence this is the change in component index related to unemployment level expectations that is proved to be the best predictor of changes in household saving and borrowing rates. Its four lags, taken on their own, explain 23, 22 and 30 % of the variation of changes in total household saving rate, voluntary household saving rate and household borrowing rate, respectively. High influence is exerted also by index, which gives the appraisal of present purchasing climate.

Expanding the set of lagged changes in fundamental variables with lagged change in consumer confidence indicator yields a positive effect at the 1-percent level by expected unemployment indicator in both household saving and borrowing models and by present purchasing climate indicator in household borrowing models. Statistically significant increase in the predictive power of household saving and borrowing models is demonstrated also in case of overall Forward Consumer Confidence Index. The highest increment to the $\bar{R}^{2}$ (of 15.5 % points) is provided by index of unemployment level expectations in household borrowing model. It is worth emphasizing that borrowing behavior seems to be more confidence sensitive than saving behavior is.

The empirical findings suggest that some consumer confidence indexes (subjective indicators) contain predictive ability beyond economic fundamentals (objective indicators) and that they are useful in analyzing and forecasting household saving and borrowing behavior. Further research on the influence of financial optimism or pessimism on household saving and borrowing behavior at the household level is recommended. Better understanding of the household financial expectations to household financial decisions relationship should be valuable input into a number of policy areas, in particular into monetary policy and financial stability analysis. Consumer confidence may serve to reinforce or counteract policy changes; therefore, it is essential for policymakers to consider it in order to improve prediction of policy effects.

## Footnotes

1 In what follows, the word “sentiment” and “confidence” are used interchangeably.
